# Parental Stress in Academic Emergency Medicine Physicians

**DOI:** 10.1111/acem.70145

**Published:** 2025-09-13

**Authors:** Deborah B. Diercks, Michelle Lall, Anne Messman, Ellen O'Connell, Meagan Hunt, Mia Karamatsu, Katie Pettit, D. Mark Courtney

**Affiliations:** ^1^ University of Texas Southwestern Medical Center Dallas Texas USA; ^2^ Emory School of Medicine Atlanta Georgia USA; ^3^ Wayne State University Detroit Michigan USA; ^4^ Wake Forest University School of Medicine Winston‐Salem North Carolina USA; ^5^ Stanford University Palo Alto California USA; ^6^ Indiana University Indianapolis Indiana USA

## Abstract

**Background:**

Recent publications have shown that women are more likely to leave emergency medicine at a younger age than men. We aim to describe the prevalence of parental stress in academic emergency medicine and its association with scheduling practices and desire to leave medicine.

**Methods:**

Blinded survey sent to eight geographically diverse academic sites. Survey included five domains: academic rank and perception of progress, child and childcare characteristics, clinical scheduling practices, plans to leave medicine, and validated psychometric measures including the Parental Stress Scale (PSS: normal population score 35–45). Likert scale responses were dichotomized as either moderate/extremely likely versus less than moderately likely/unsure. Descriptive statistics were calculated, and linear and multivariate regression analyses were performed using STATA 16.

**Results:**

A total of 280 surveys were accessed, and 225 (80%) surveys had PSS completed. Of this cohort, there were 90 females, 123 men, 1 intersex, and 15 surveys had no sex reported. The median number of children was 2 (IQR 1–3), and the median age of the youngest child was 4 (IQR 1–9). The parental stress scale median score was 40 (IQR 35–46). There was no significant difference in the parental stress scale by sex. The number of children (B‐coeff −1.88, *p* = 0.007), age of the youngest child (B‐coeff −4.2, *p* = 0.000), use of daycare (B‐coeff 3.8, *p* = 0.027), ability to preference times of shifts (day, swing, night shift) (B‐coeff −2.4, *p* = 0.046), being a nocturnist (B‐coeff 2.75, *p* = 0.006), and being able to completely set their own schedule in terms of days and times worked (B‐coeff −2.19, *p* = 0.03) were associated with the PSS score. The parental stress scale was not associated with the likelihood to leave emergency medicine or leave the current job in 5 years.

**Conclusion:**

Academic emergency physicians had parental stress scale scores similar to the general population. Parental stress scale score was not associated with a plan to leave emergency medicine.

## 1 Introduction

Emergency medicine (EM) physicians have one of the highest rates of burnout among any physician group [1]. It has been postulated that this is related to the workplace environment, variable schedules, and challenging work‐life integration. This elevated stress level in EM compared with other specialties has been reported to start as early as residency. One study noted that EM residents reported a stress level of 80.5% compared to levels below 75% in internal medicine and family medicine [2]. In addition, studies have reported EM physicians self‐reporting high rates of psychological stress associated with feeling overstretched, with an impact on family life and high rates of work–family conflict [3, 4]. In general, EM physicians have a high rate of home life anxiety and overall life stress [5].

Recent studies have evaluated the EM workforce, noting significant differences in attrition rates between men and women. In addition, it has been noted that women leave academic emergency medicine at a younger age than men. Prior studies outside of EM have reported that shift work may also increase the level of family stress. This has been attributed to rotating schedules, unreliable childcare as a result of variable scheduling, and poor sleep quality [6, 7]. In addition, there have been studies that report flexible scheduling as a factor that may mitigate levels of stress [8].

The Parental Stress Scale (PSS) is a validated measure of parental stress that has been studied in and outside of medicine. It is an 18‐item self‐reported measure with a total score range from 18 to 90, with higher scores indicating higher levels of parental stress [9]. Studies have reported a PSS score of 38.1 (SD 9.4) among mothers, 35.2 (SD 9.1) among parents of children with leukemia, and 47.8 (SD 10.1) among parents of children aged 6–11 years with behavioral issues [10]. Although this scale has not been specifically studied in emergency medicine, it has been evaluated in resident and fellow trainees and showed a mean score of 44.3 (SD 12.3) [11]. Our study aimed to assess the prevalence and level of parental stress among academic emergency physicians. Importantly, we sought to determine whether there is an association between parental stress and desire to leave emergency medicine as well as to identify potential mitigating factors. It is possible that by understanding the dimensions of stress captured by the PSS, healthcare systems can design support systems and implement policies aimed at improving work‐life integration for EM physicians.

## 2 Methods

### 2.1 Study Design and Participants

Participants were recruited via a direct email with information on the study that came from study champions at nine academic institutions. The emailed information included a description of the inclusion criteria as that of being a parent with a child aged ≤ 18 years old and being willing to participate via a single one‐time survey that would be anonymized. This study is IRB‐approved.

### 2.2 Measures

The survey included a series of standardized and validated instruments to measure psychosocial and stress‐related outcomes. Participants were invited to participate and asked to provide self‐reported information via an online data collection instrument that included basic demographic information, childcare practices, type of clinical practice site, and categorical information describing their individual and group shift scheduling practices [12, 13, 14, 15, 16, 17]. They were asked to complete questions contained in the PSS, the Patient‐Reported Outcomes Measurement Information System (PROMIS) Emotional Distress‐Anxiety Short form, and the PROMIS General Life Satisfaction survey, all of which are existing, validated data collection instruments. None of these tools were derived to detect mental illness. The survey was piloted by 20 individuals across all sites to ensure validity and comprehension of questions.

Participants provided informed consent electronically before beginning the survey. The survey was administered via REDCap, ensuring data security and anonymity. It took approximately 15–20 min to complete.

### 2.3 Data Analysis

Descriptive statistics were calculated for demographic and survey variables. PROMIS T‐scores were computed using the scoring manuals and software provided by the PROMIS Assessment Center. PSS scores were calculated by summing item responses, with reverse coding applied to positive items as per the scoring guidelines. Descriptive analysis was performed, presenting 95% confidence intervals, medians/means, and odds ratios when relevant. Nonparametric tests such as Kruskal–Wallis and Wilcoxon rank sum were performed. Statistical analyses, including correlational and regression analyses, were conducted using Stata 16, College Station.

## 3 Results

The redcap survey was accessed by 311 academic faculty, with 280 agreeing to participate (23 did not meet inclusion criteria). The cohort included 158 males (56%) and 116 females (44%) with 78% reporting race as Caucasian, 5% Black or African American, 18% Asian, and 4.3% Hispanic. Mean age was reported as 43 (SD 23) years. Median number of clinical hours worked was 88 h a month, and this did not differ by sex (*p* = 0.84). Academic rank is shown in Table 1, with the majority of participants being Assistant Professors. Additionally, academic rank did not vary by sex (*p* = 0.67). Females were more likely than males to self‐report a perception that their pursuit of promotion has been prolonged because they are a parent (OR 2.88 (95% CI 1.64–5.1)). However, we also asked respondents to describe their perception of their own career progress, and when comparing the proportion who described career progress as “Excellent” or “Good” by sex, there was no significant difference.

**TABLE 1:** Demographics.

	*n* (%)
Sex	
Female	116 (43.4%)
Male	150 (56.2%)
Intersex	1 (0.4%)
Ethnicity	
Hispanic or Latino	12 (4.3%)
NOT Hispanic or Latino	262 (93.6%)
Unknown/Not Reported	6 (2.1%)
Race	
Asian	30 (10.8%)
Black or African American	13 (4.7%)
White	217 (78.1%)
More than one race	6 (2.2%)
Unknown/Not reported	12 (4.3%)
Age (years)	41 (IQR 37–46)
What is your academic rank?	
Instructor/Fellow	20 (7.8%)
Assistant Professor	134 (52.5%)
Associate Professor	67 (26.3%)
Professor	34 (13.3%)
Do you believe your pursuit of promotion has been prolonged by your role as a parent?	
No	133 (52.4%)
Yes	121 (47.6%)
Have you reduced your FTE due to family related reasons within the last 5 years?	
No	192 (78.7%)
Yes	52 (21.3%)
What is your perception of how you are progressing on your academic goals?	
No progress	31 (12.2%)
Some progress	73 (28.7%)
Moderate progress	64 (25.2%)
Good progress	55 (21.7%)
Excellent progress	31 (12.2%)
Are you currently the primary financial supporter of the family?	
No	58 (24.6%)
Yes	178 (75.4%)
Your age when your first child was born	32 (IQR 30–35)
How many children do you have?	2 (IQR 2–3)
Age of your youngest child	4 (1–9)
What is your current partnership status?	
Married couple, both parents in same household	169 (69.3%)
Married couple, parents live apart > 25% of the year (military deployment, travel for work, jobs in different cities)?	3 (1.2%)
Married couple, full‐time working spouse	38 (15.6%)
Married couple, part‐time working spouse	9 (3.7%)
Married couple, stay‐at‐home spouse	15 (6.1%)
Non‐married, cohabitating partners	2 (0.8%)
Single parent	3 (1.2%)
Divorced, > 50% custody	4 (1.6%)
Blended family (a family consisting of a couple and their children from this and all previous relationships)?	1 (0.4%)

In terms of family life, over half of the participants reported being married with both parents in the household (69.2%). The median number of children was 2, with the median age of the youngest child of 4 (IQR 1–9). Most of the participants identified themselves as the primary financial supporter of the family (75.4%). Females were less likely to identify as primary financial supporters: males 86% and females 60% (diff −26%, 95% CI −38 to −14). Having a parent take care of children (defined as any parent) (33.5%) and daycare (28.5%) were the major sources of childcare utilized. Females were more likely than males to reduce their FTE for family‐related reasons: males 14% and females 32% (diff 18%, 95% CI 6–28).

An important finding of this report is how clinical shift scheduling was perceived by respondents (Table 2). The majority noted that when filling out schedule requests, they have the ability to specify shifts that they prefer to work (57%, 95% CI 51–64) and preference for particular entire days that they prefer to work (60.4%, 95% CI 54–67) but a nontrivial percent of participants had the ability to set the days of the week that they work (28%, 95% CI 22–34). Of the cohort, 83.7% (95% CI 78–88) and 68.6% (95% CI 62–74) report it being extremely or moderately easy to trade shifts or get coverage for sick calls, respectively. In addition, 52.5% (95% CI 50–59) reported significant or complete schedule autonomy. There was no significant difference in shift scheduling practices by sex.

**TABLE 2:** Schedule process.

	*n* (%)
Does your group use a scheduling software program?	
No	4 (1.7%)
Yes	236 (98.3%)
Can you preference the types of shifts you work (example: day or night)?	
No	103 (42.7%)
Yes	138 (57.3%)
Can you preference days of the week you work?	
No	95 (39.6%)
Yes	145 (60.4%)
Do you have the ability to provide unlimited requests?	
No	150 (62.5%)
Yes	90 (37.5%)
Can you set the days of the week you work?	
No	173 (72.1%)
Yes	67 (27.9%)
Are you a nocturnist?	
No	199 (83.3%)
Yes	40 (16.7%)
How likely is your group to trade shifts if asked?	
Extremely unlikely	3 (1.2%)
Moderately unlikely	33 (13.6%)
Unsure	5 (2.1%)
Moderately likely	135 (55.8%)
Extremely likely	66 (27.3%)
How difficult is it to get emergency coverage for your shifts?	
Extremely difficult	16 (6.6%)
Moderately difficult	40 (16.5%)
Unsure	20 (8.3%)
Moderately easy	124 (51.2%)
Extremely easy	42 (17.4%)
What is your perception of your scheduling autonomy?	
No autonomy or input	1 (0.4%)
Minimal input	20 (8.3%)
Some input	93 (38.8%)
Significant input on the days and times worked	112 (46.7%)
Complete schedule control	14 (5.8%)

The median PSS score was 40 (IQR 35–46). There was no significant difference by sex *p* = 0.36. However, all of the following were independently associated with the PSS on univariate linear regression: number of children (B‐coeff −1.88, *p* = 0.007), age of youngest child (B‐coeff −4.2, *p* = 0.000), use of daycare (B‐coeff 3.8, *p* = 0.027), ability to preference times of shifts (day, swing, night shift) (B‐coeff −2.4, *p* = 0.046), being a nocturnist (B‐coeff 2.75, *p* = 0.006), and being able to completely set their own schedule in terms of days and times worked (B‐coeff −2.19, *p* = 0.03). There was no association between PSS scores and number of clinical hours, sex, being the primary financial supporter of the family, partner engagement in the raising of children, ability to preference days, willingness to trade shifts, and coverage of sick call. When adjusting for significant covariates on multivariable linear regression, only the age of the youngest child (B‐coeff −0.33, *p* = 0.011) and being a nocturnist (B‐coeff 4.96, *p* = 0.002) were independently associated with higher PSS scores. In addition, in a linear regression adjusting for sex, a perception of low partner engagement was associated with a higher PSS score (B‐coeff −1.82, *p* = 0.049) in a separate analysis. There was a weak correlation between the PROMIS anxiety score (*r* = 0.34) and the PSS score, and a moderate inverse correlation between the PROMIS general life satisfaction score and the PSS score (*r* = −0.59).

In the 242 participants that responded, 22.7% (95% CI 17.6–28.5) and 14% (95% CI 10–19) reported being extremely or moderately likely to leave their job or medicine within the next 5 years, respectively (Table 3). The PSS score was not associated with the likelihood of leaving a job or medicine, *p* = 0.09 and *p* = 0.479, respectively. The PROMIS anxiety score and PROMIS general life satisfaction score were independently associated with the likelihood of leaving their job and leaving medicine. We did univariate analysis for a variety of variables for the outcome of reporting extremely/moderately likely to leave a job. Results and OR are as follows: deferral of promotion (OR 2.3, 95% CI 1.18–4.54), ability to preference specific shifts (OR 0.48, 95% CI 0.25–0.94), ease of shift trades (OR 0.43, 95% CI 0.19–0.96), ease of sick call coverage (OR 0.37, 95% CI 0.28–0.81), and perception of high level of schedule autonomy (OR 0.52, 95% CI 0.0–0.73). There was no association between likelihood to leave a job by sex, career progression perception, number of children, age of youngest child, ability to preference specific days worked, ability to set the days of the week worked, and clinical hours worked.

**TABLE 3:** Likelihood to leave.

	*N* (%)
What is the likelihood you will leave your clinical job in 5 years?	
Extremely unlikely	93 (38.4%)
Moderately unlikely	61 (25.2%)
Unsure	33 (13.6%)
Moderately likely	33 (13.6%)
Extremely likely	22 (9.1%)
What is the likelihood will leave the field of medicine in 5 years?	
Extremely unlikely	132 (54.5%)
Moderately unlikely	48 (19.8%)
Unsure	28 (11.6%)
Moderately likely	24 (9.9%)
Extremely likely	10 (4.1%)

## 4 Discussion

Given the high attrition of EM physicians out of medicine and some data that suggest attrition is greatest in females, we aimed to describe parental stress among EM physicians and whether sex was associated with high stress levels [18, 19, 20, 21]. The hypothesis was that perhaps parental stress was one driver of attrition in emergency medicine. In this multicenter study of academic physicians, parental stress levels as measured by the PSS score did not differ by sex. In addition, the quantitative value of the PSS score was similar to other studies, suggesting that EM physicians do not have a higher amount of parental stress than other cohorts [11]. There was, however, an association between the number of children and the age of the youngest child with the PSS. Parents with more children and younger children had higher PSS scores.

There have been studies that evaluate the impact of work‐life integration and burnout in physicians. Some studies have shown no impact on parental status and burnout. In a study of German resident physicians, parental stress was not associated with burnout [22]. However, in the Maslach Burnout Inventory emotional exhaustion scale, parents had higher levels of emotional exhaustion. It should be noted that different countries may have different policies regarding parental leave, which may impact parental stress. In addition, this study found work‐life conflict was associated with burnout [22]. Studies evaluating surgeons report that parenting is a significant threat to the pipeline of female surgeons, with childcare needs and conflicting schedules driving many physician mothers to seek part‐time work [23]. Other studies report that women leaving their employment at an academic institution noted work‐life balance as a key theme associated with leaving academic medicine [24]. Within emergency medicine, a study of female faculty and residents at eight academic medical institutions assessed career advancement. Faculty respondents identified childbearing/child‐rearing as a reason for stunting career growth [25].

Our study noted no difference in parental stress levels by sex in a univariate analysis. However, some findings were consistent with prior work. The use of daycare was associated with parental stress in a univariate analysis but was not significant when other factors were adjusted for. The association between parental stress and daycare may be related to the lack of flexibility in the hours of care provided, which certainly impacts EM physicians due to the variability in schedules [13]. Our finding that the age of the youngest child is independently associated with parental stress levels is also consistent with prior studies showing parental leave and return to work barriers have been noted as problematic areas in medicine [26].

Our study also had some unique demographics that may have impacted our findings. Prior studies have shown that being a single parent and one's family structure impact parental stress [14, 27]. Our cohort was almost entirely comprised of married physicians. This may have reduced the parental stress level overall. In addition, 89.5% reported significant partner engagement in raising children. This level of support may reduce the overall level of PSS, although not statistically significant in our analysis unless sex was adjusted for. Another unique finding in our study was the lack of association between schedule uncertainty and the PSS score. Prior studies have shown that nonstandard work schedules are associated with higher levels of parental stress [16, 28]. In our study, a nonstandard shift schedule and ability to preference shifts were inversely associated with the PSS score but were not independently associated with adjustments for other factors such as the age of the youngest child. This highlights the unique challenges that parents of young children face in terms of balancing work‐life responsibilities.

A secondary aim of this study was to determine whether there was an association between parental stress and attrition in emergency medicine. Prior studies have shown that women leave emergency medicine 10 years earlier than men [19]. Our study found no association with PSS score and a moderate‐to‐high likelihood of leaving emergency medicine or medicine within 5 years. However, we also assessed two PROMIS measures: anxiety and satisfaction with life. We noted that there was only a weak association between PSS score and PROMIS anxiety score and a moderate inverse correlation with the PROMIS general life satisfaction score. However, both of the PROMIS scores were associated with the likelihood of leaving the job/emergency medicine within 5 years. In addition, lack of schedule control and lower group support for shift coverage appeared to be associated with the likelihood of leaving an academic job. This highlights the impact of scheduling on career satisfaction in emergency medicine.

Our study has several limitations. There were a large number of participants who did not complete the survey or specific questions on the survey. This not only limits the ability to look at interactions but also raises concerns about nonresponse bias in results. We had a high percentage of married participants in the cohort, which may have biased the results. Survey recipients experiencing significant parental stress may not have even considered participating in a 15‐ to 20‐min survey. In addition, our cohort lacks substantial diversity. This may have masked unique stressors that exist for minoritized individuals. It is possible that parental stress could still be a factor for EM attrition, as shown in previous literature, but that it was not shown through our specific choice of measurement (PSS).

In conclusion, we report that the PSS scores in emergency medicine physicians are similar to prior studies of general populations and did not differ by sex. The PSS score was not associated with the likelihood of leaving emergency medicine.

## Author Contributions

D.B.D. and D.M.C. conceived the idea. D.B.D., M.L., A.M., E.O.C., M.H., M.K., and K.P. developed and edited the survey tool. D.B.D., M.L., A.M., E.O.C., M.H., M.K., and K.P. reviewed the data. All authors edited the manuscript.

## Conflicts of Interest

D.B.D.: institutional research funding: Tosoh, Siemens, Abbott. Editorial Boards: Circulation and Academic Emergency Medicine. Board of Directors: ABEM and Emergencies in Medicine. D.M.C.: Editorial Board: Academic Emergency Medicine. ML: Board of Directors Society of Academic Emergency Medicine. A.M., E.O., M.K., M.H., K.P.: none.

## Data Availability

The data that support the findings of this study are available upon request from the corresponding author. The data are not publicly available due to privacy or ethical restrictions.
